# The accuracy of radiomics in diagnosing tumor deposits and perineural invasion in rectal cancer: a systematic review and meta-analysis

**DOI:** 10.3389/fonc.2024.1425665

**Published:** 2025-01-08

**Authors:** Xuewu Liu, Feng Lin, Danni Li, Nan Lei

**Affiliations:** Radiology Department, The People’s Hospital of Lezhi, Ziyang, Sichuan, China

**Keywords:** machine learning, rectal cancer, perineural invasion, tumor deposits, systematic review, meta-analysis

## Abstract

**Background:**

Radiomics has emerged as a promising approach for diagnosing, treating, and evaluating the prognosis of various diseases in recent years. Some investigators have utilized radiomics to create preoperative diagnostic models for tumor deposits (TDs) and perineural invasion (PNI) in rectal cancer (RC). However, there is currently a lack of comprehensive, evidence-based support for the diagnostic performance of these models. Thus, the accuracy of radiomic models was assessed in diagnosing preoperative RC TDs and PNI in this study.

**Methods:**

PubMed, EMBASE, Web of Science, and Cochrane Library were searched for relevant articles from their establishment up to December 11, 2023. The radiomics quality score (RQS) was used to evaluate the risk of bias in the methodological quality and research level of the included studies.

**Results:**

This meta-analysis included 15 eligible studies, most of which employed logistic regression models (LRMs). For diagnosing TDs, the c-index, sensitivity, and specificity of models based on radiomic features (RFs) alone were 0.85 (95% CI: 0.79 - 0.90), 0.85 (95% CI: 0.75 - 0.91), and 0.82 (95% CI: 0.70 - 0.89); in the validation set, the c-index, sensitivity, and specificity of models based on both RFs and interpretable CFs were 0.87 (95% CI: 0.83 - 0.91), 0.91 (95% CI: 0.72 - 0.99), and 0.65 (95% CI: 0.53 - 0.76), respectively. For diagnosing PNI, the c-index, sensitivity, and specificity of models based on RFs alone were 0.80 (95% CI: 0.74 - 0.86), 0.64 (95% CI: 0.44 - 0.80), and 0.79 (95% CI: 0.68 - 0.87) in the validation set; in the validation set, the c-index, sensitivity, and specificity of models based on both RFs and interpretable CFs were 0.83 (95% CI: 0.77 - 0.89), 0.60 (95% CI: 0.48 - 0.71), and 0.90 (95% CI: 0.84 - 0.94), respectively.

**Conclusions:**

Diagnostic models based on both RFs and CFs have proven effective in preoperatively diagnosing TDs and PNI in RC. This non-invasive method shows promise as a new approach.

**Systematic review registration:**

https://www.crd.york.ac.uk/prospero/display_record.php?RecordID=498660, identifier CRD42024498660.

## 1 Introduction

Rectal cancer (RC) is a common tumor in clinical practice worldwide and a significant contributor to cancer-related mortality (1, 2). According to the Global Cancer Statistics 2020, colorectal cancer currently ranks third among all tumors in terms of new cases and second in terms of mortality (2). In the past, the prognosis of RC was primarily determined by the depth of tumor infiltration, the presence of lymph node metastasis, and the potential for distant metastasis. However, as the understanding of RC gradually deepens, tumor deposits (TDs) and perineural invasion (PNI) have also become evaluation indicators.

TDs are focal masses of adenocarcinoma or discontinuous extramural extensions that are found in the perirectal region. They lack vascular/neural structures or residual lymph nodes on histological examination (3). According to the 8th edition of the TNM staging system developed by the American Joint Commission on Cancer, any T lesion with negative regional lymph node metastasis and positive TDs is categorized as N1c (4). The presence of positive TDs can enhance the clinical staging of RC patients and affect subsequent clinical treatment plans. PNI is a biological process whereby cancer cells invade nerves and spread along the nerve sheath (5). Uccello et al. (6) discovered in animal studies that radiation therapy could exacerbate the activity of PNI-positive RC. In addition, studies have shown that PNI can serve as an indicator for distinguishing whether patients can benefit from neoadjuvant chemoradiotherapy (nCRT) and postoperative adjuvant chemotherapy (7, 8). Therefore, accurate and objective preoperative diagnosis of TDs and lymph node metastasis is of great significance for the personalized treatment of patients.

The radiomics method, a recently developed non-invasive evaluation technique, has received widespread attention from investigators. By applying a variety of mathematical algorithms to extract numerous fine-grained features from medical images, perform quantitative analysis, and create useful disease diagnosis and prognosis models to aid in individualized clinical decision-making, radiomics disrupts the conventional analytical framework of imaging (9). In recent years, radiomics studies on RC have included preoperative staging (10), prediction of metastasis (11), prediction of radiotherapy and chemotherapy efficacy (12), and prediction of survival (13). These studies demonstrated the analytical and predictive ability of radiomics for RC. Some investigators have explored the accuracy of radiomics in the preoperative detection of TDs and PNI. However, a comprehensive evidence-based medicine study is currently lacking to support this claim. Therefore, this systematic review and meta-analysis was performed to close this gap and facilitate the application of radiomics in this field.

## 2 Methods

### 2.1 Eligibility criteria

#### 2.1.1 Inclusion criteria

1. The subjects were individuals who had been diagnosed with RC through pathological examination.
2. Investigators developed or used machine learning models (MLMs) that included at least image features to diagnose preoperative TDs or PNI.
3. The article was written in English.
4. The investigators clearly described the radiomics models and reported the diagnostic performance indicators of the models, or provided enough data for inferring the c-index and/or accuracy.

#### 2.1.2 Exclusion criteria

1. The study types were meta, review, guidelines, expert opinions, etc.
2. The investigators only conducted differential factor analysis, and did not construct a complete MLM.
3. The investigators did not describe the modeling process or methods, and/or did not report the results of model performance measurements.
4. The investigators only studied image segmentation.

### 2.2 Data sources and search strategies

PubMed, EMBASE, Web of Science, and Cochrane Library were searched for relevant articles published up to December 11, 2023. The search adopted a combination of subject-specific and non-specific terms without any limitations on publication area or period. Additionally, a manual search of the references of included articles was performed for potential eligible studies. The search procedures and strategies are detailed in **Supplementary Table S1**.

### 2.3 Study screening and data extraction

After importing the articles into Endnote, any duplicates were removed at first. Then, the titles or abstracts were read to select those meeting the specified criteria. Finally, the full texts were downloaded and read to select eligible studies. Prior to data extraction, a standard data extraction spreadsheet was developed. It included a range of information, such as the first author, publication year, country, study type, patient source, diagnostic purpose, gold standard for target events, tumor staging, image source, image parameter description, number of investigators, ROI segmentation software, total number of events, and total number of cases. The testing and validation sets encompassed the number of cases and events, as well as variable screening methods, model types, and modeling variables.

Two investigators independently conducted the study screening and data extraction, which were cross-checked. Any objections were addressed through negotiation with a third reviewer.

### 2.4 Assessment of study quality

Two investigators used the radiomics quality score (RQS) (14) to assess the risk of bias in the methodological quality and research level of the included studies, and conducted cross-checks. Any differences resolved by the third reviewer.

### 2.5 Outcomes

The outcome indicators of this study mainly included the c-index for reflecting the overall accuracy of the model, and the sensitivity and specificity for identifying the accuracy of outcome events.

### 2.6 Synthesis methods

This systematic review and meta-analysis was conducted on the indicator (c-index) used to reflect the overall accuracy of radiomic models. In instances where the 95% confidence interval (CI) and standard error were not provided for this indicator, its standard error was estimated using the study by Debray et al. (15). Considering the differences in variables and inconsistent parameters among MLMs, a random-effects model was used to conduct the meta-analysis on c-index. Furthermore, a bivariate mixed effects model was adopted for the meta-analysis on sensitivity and specificity. In this study, a diagnostic four-grid table was used for analyzing sensitivity and specificity. However, the diagnostic four-grid table was not provided in most original studies. In this case, two approaches were applied to create the table. The first approach involved using sensitivity, specificity, precision, and the number of cases for calculation purposes. The second approach involved extracting sensitivity and specificity based on the best Youden index and then calculating based on the number of cases. This study was implemented using R version 4.2.0 (R development Core Team, Vienna, http://www.R-project.org).

## 3 Results

### 3.1 Study screening

**Figure 1:**
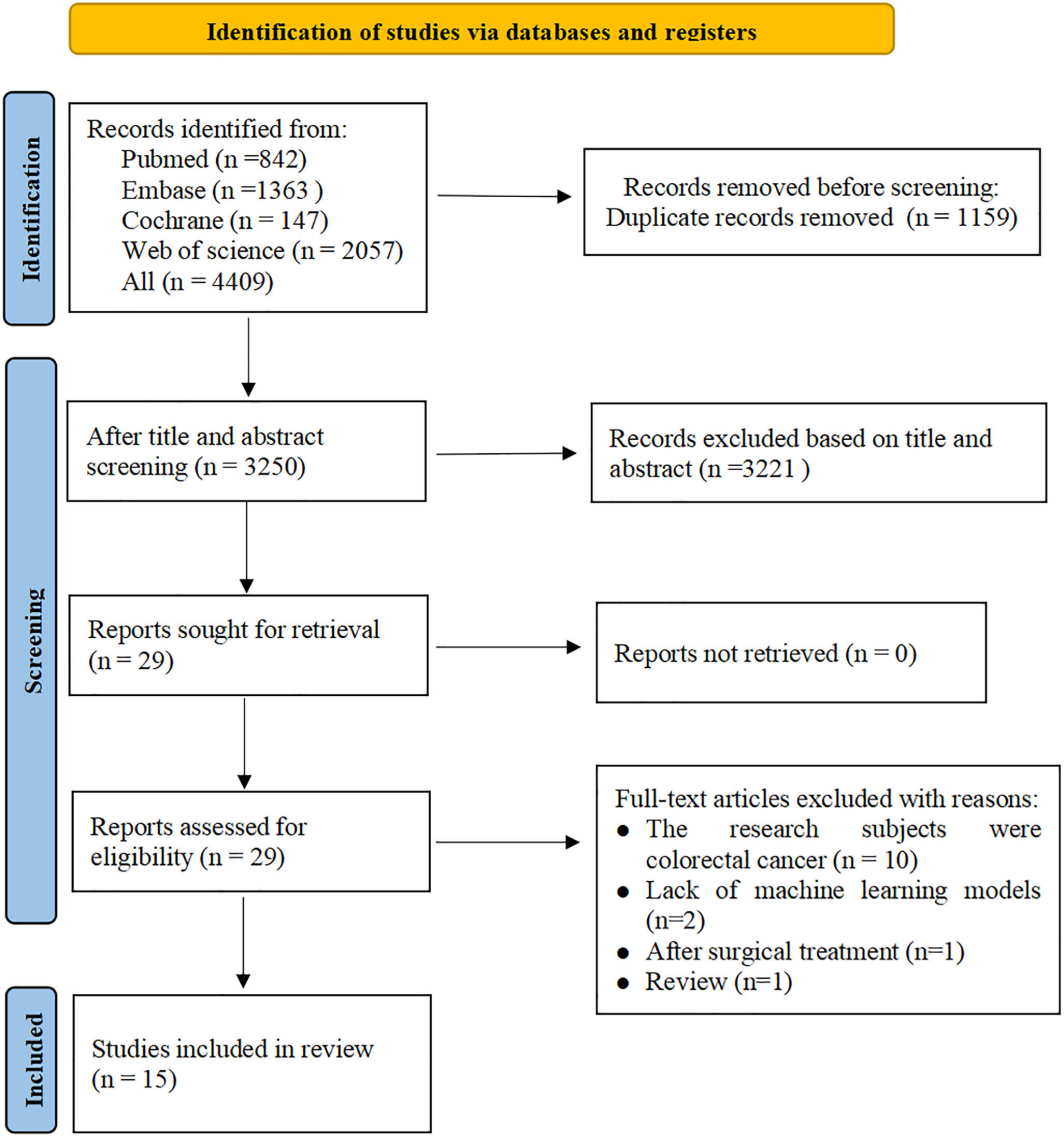
Literature screening process.

A total of 4,409 studies were retrieved from four databases, with 3,250 remaining after removing duplicates. After reading the titles and abstracts, 3,220 studies were excluded. After reading the full text of the remaining 29 studies, 14 were excluded for the reasons outlined in **Figure 1**. Finally, a total of 15 studies (16–30) were included.

### 3.2 Study characteristics

For PNI, all studies were conducted in China and published between 2020 and 2021. Three studies utilized MRI (16–18), one study employed CT (19), and one study utilized both CT and MRI (20). Four studies had two investigators (17–20), while one study had three investigators (16). The ITK-SNAP software was utilized in all four studies (16–18, 20), except for one study that did not specify the software utilized for image segmentation (19). All studies employed the Least Absolute Shrinkage and Selection Operator (LASSO) method for RF selection and logistic regression modeling. Meanwhile, all studies constructed radiomics scores and developed models together with clinical features. All studies were retrospective and included 938 subjects, of whom 305 were PNI-positive.

For TDs, 9 studies were conducted in China (21–29), and 1 in the United States (30). Eight studies utilized MRI images (21–25, 27, 28, 30), 1 employed CT (26), and 1 combined MRI and US (29). Eight studies had two investigators (21–23, 25, 26, 28–30), while two studies had three investigators (24, 27). In the image segmentation stage, the ITK-SNAP software was utilized in 5 studies (23–25, 27, 28), while 3D slicer software, A.K software, UltrasomicsPlatform, and TexRAD software were employed in other studies (21, 22, 29, 30), and 1 study did not specify the segmentation software adopted (26). Eight studies utilized the LASSO method for RF selection (22–29), one study applied the Principal Component Analysis (PCA) method (21), and one study did not specify the method for feature selection (30). Seven studies employed LRMs (21–23, 26–28, 30), 1 applied the support vector machine model (25), 1 adopted deep learning model (29), and 1 utilized both the deep learning model and the support vector machine model (24). Five studies constructed radiomics scores and carried out model construction together with clinical features (21–23, 27, 28). There were 8 retrospective studies (21, 23–28, 30) and 2 prospective studies (22, 29), which included a total of 2,002 subjects, with 648 patients having TDs.

The study characteristics are presented in **Table 1**.

**Table 1:** Characteristics of included studies.

Study	Country	Study design	Data source	Diagnostic purpose	Stage	Image source	Number of patients in the training set	Number of patients in the test set	Types of machine learning	Technique used for feature selection
Yan-song Yang (2021) (16)	China	Retro	Single institution	PNI	I-III	MRI	99	41	LR	LASSO
Yang Zhang (2022) (17)	China	Retro	Single institution	PNI	I-III	MRI	194	85	LR	LASSO
Yu Guo (2021) (20)	China	Retro	Single institution	PNI	I-III	MRI, CT	65	29	LR	LASSO
Jiayou Chen (2020) (18)	China	Retro	Single institution	PNI	I-III	MRI	87	35	LR	LASSO
Mou Li (2021) (19)	China	Retro	Single institution	PNI	I-III	CT	242	61	LR	LASSO
Rui Yang (2023) (21)	China	Retro	Single institution	TDS	T3	MRI	114	49	LR	PCA
Hang Li (2023) (22)	China	Pros	Multiple institution	TDS	I-III	MRI	94	114 (92 Exception Verification)	LR	LASSO
Yumei Jin (2023) (23)	China	Retro	Single institution	TDS	I-III	MRI	163	41	LR	LASSO
Chunlong Fu (2023) (24)	China	Retro	Single institution	TDS	I-III	MRI	133	NA	SVM, ANN	LASSO
Feiwen Feng (2023) (25)	China	Retro	Single institution	TDS	I-III	MRI	103	45	SVM	LASSO
Yong-Chang Zhang (2022) (26)	China	Retro	Single institution	TDS	I-III	CT	163	56 (15 additional verification)	LR	LASSO
Yan-song Yang (2021) (27)	China	Retro	Single institution	TDS	I-III	MRI	98	41	LR	LASSO
Yumei Jin (2021) (28)	China	Retro	Single institution	TDS	I-III	MRI	203	51	LR	LASSO
Isha D. Atre (2021) (30)	USA	Retro	Single institution	TDS	I-III	MRI	15	NA	LR	NA
Li-Da Chen (2019) (29)	China	Pros	Single institution	TDS	I-III	US, MRI	87	40	ANN	LASSO

Retro, Retrospective; Pros, Prospective; LR, Logistic regression; SVM, Support vector machine; ANN, Artificial neural network; LASSO, Least Absolute Shrinkage and Selection Operator; PCA, Principal component analysis; NA, Not available.

### 3.3 Assessment of study quality

All studies described detailed scanning protocols and used multiple segmentation methods to segment images. Of the studies reviewed, 11 reported cut-off values, while 9 reported calibration statistics. Fourteen studies had validation sets or conducted self-cross validation. However, one study did not have these elements in place, resulting in a deduction of 5 points for this study. None of the studies provided open-source data and code. The average score for all studies is 10.93 points.

### 3.4 Meta-analysis

#### 3.4.1 Tumor deposits

##### 3.4.1.1 Synthesized results

**Figure 2:**
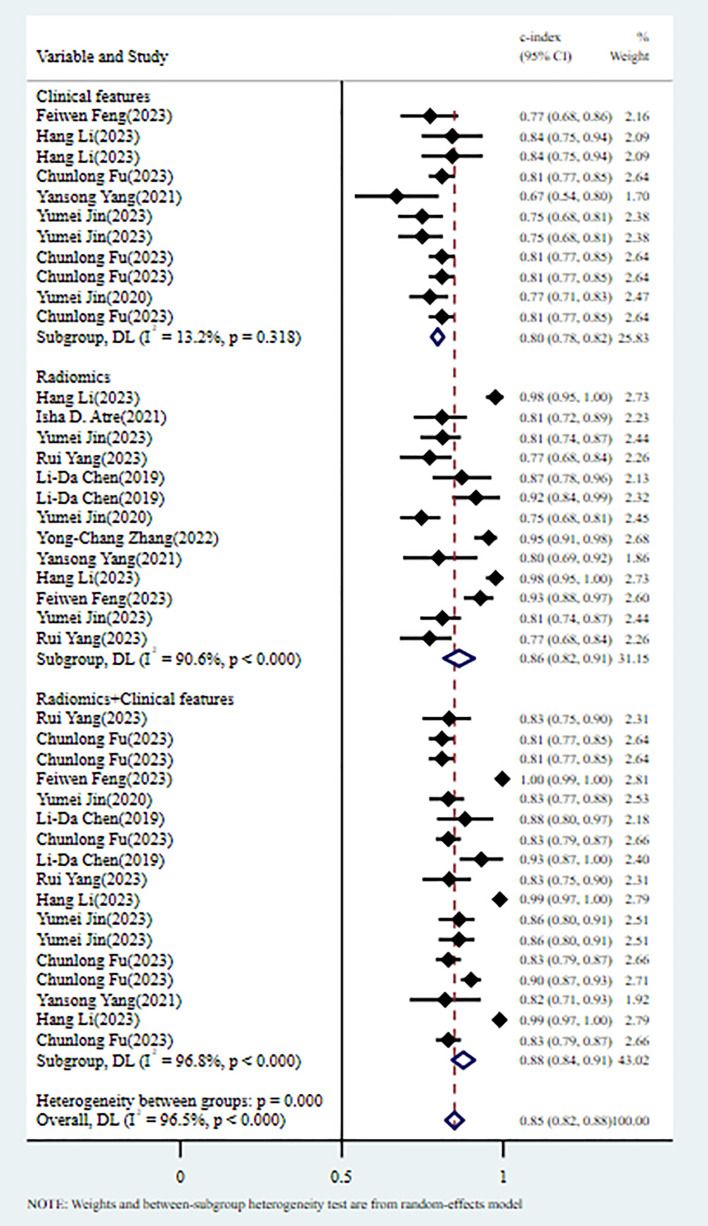
C-index meta-analysis forest diagram for diagnosing TDs using machine learning models in the training set.

In the training set, 13 MLMs were constructed based on radiomic features (RFs) alone. The c-index, sensitivity, and specificity, as summarized by a random-effects model, were 0.86 (95% CI: 0.82 - 0.91), 0.83 (95% CI: 0.76 - 0.88), and 0.85 (95% CI: 0.74 - 0.91), respectively. Eleven MLMs were constructed based solely on clinical features (CFs). A random-effects model was used, and the pooled c-index, sensitivity, and specificity were 0.80 (95% CI: 0.78 - 0.82), 0.84 (95% CI: 0.73 - 0.91), and 0.76 (95% CI: 0.70 - 0.80), respectively. Seventeen MLMs were constructed based on RFs combined with interpretable CFs; a random-effects model was used, and the pooled c-index, sensitivity, and specificity were 0.88 (95% CI: 0.84 - 0.91), 0.91 (95% CI: 0.85 - 0.95), and 0.82 (95% CI: 0.74 - 0.88), respectively. The forest maps of c-index and the sensitivity and specificity obtained from different modeling variables are shown in **Figure 2** and **Supplementary Figures S1**-**S3**.

**Figure 3:**
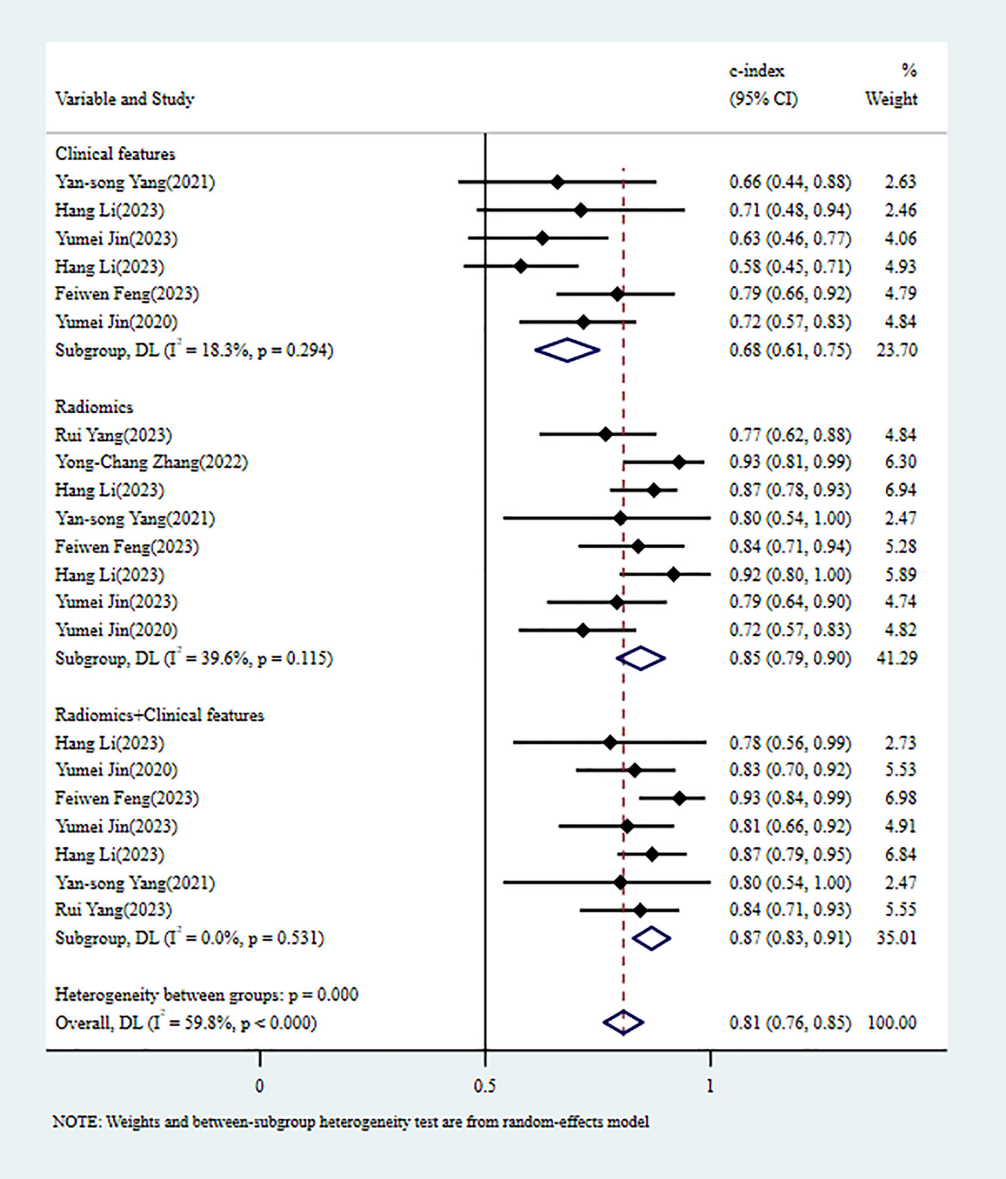
C-index meta-analysis forest diagram for diagnosing TDs using machine learning models in the validation set.

In the validation set, 8 MLMs were constructed based on RFs alone. The c-index, sensitivity, and specificity, as summarized using a random-effects model, were 0.85 (95% CI: 0.79 - 0.90), 0.85 (95% CI: 0.75 - 0.91), and 0.82 (95% CI: 0.70 - 0.89), respectively. Six MLMs were constructed based solely on CFs; a random-effects model was used, and the pooled c-index, sensitivity, and specificity were 0.68 (95% CI: 0.61 -0.75), 0.48 (95% CI: 0.28 - 0.69), and 0.79 (95% CI: 0.69 - 0.86), respectively. Seven MLMs were developed based on RFs combined with interpretable CFs; a random-effects model was used, and the pooled c-index, sensitivity, and specificity were 0.87 (95% CI: 0.83 - 0.91), 0.91 (95% CI: 0.72 - 0.99), and 0.65 (95% CI: 0.53 - 0.76), respectively. The forest maps of c-index and the sensitivity and specificity obtained from different modeling variables are shown in **Figure 3** and **Supplementary Figures S4**-**S6**.

##### 3.4.1.2 Reporting biases

**Figure 4:**
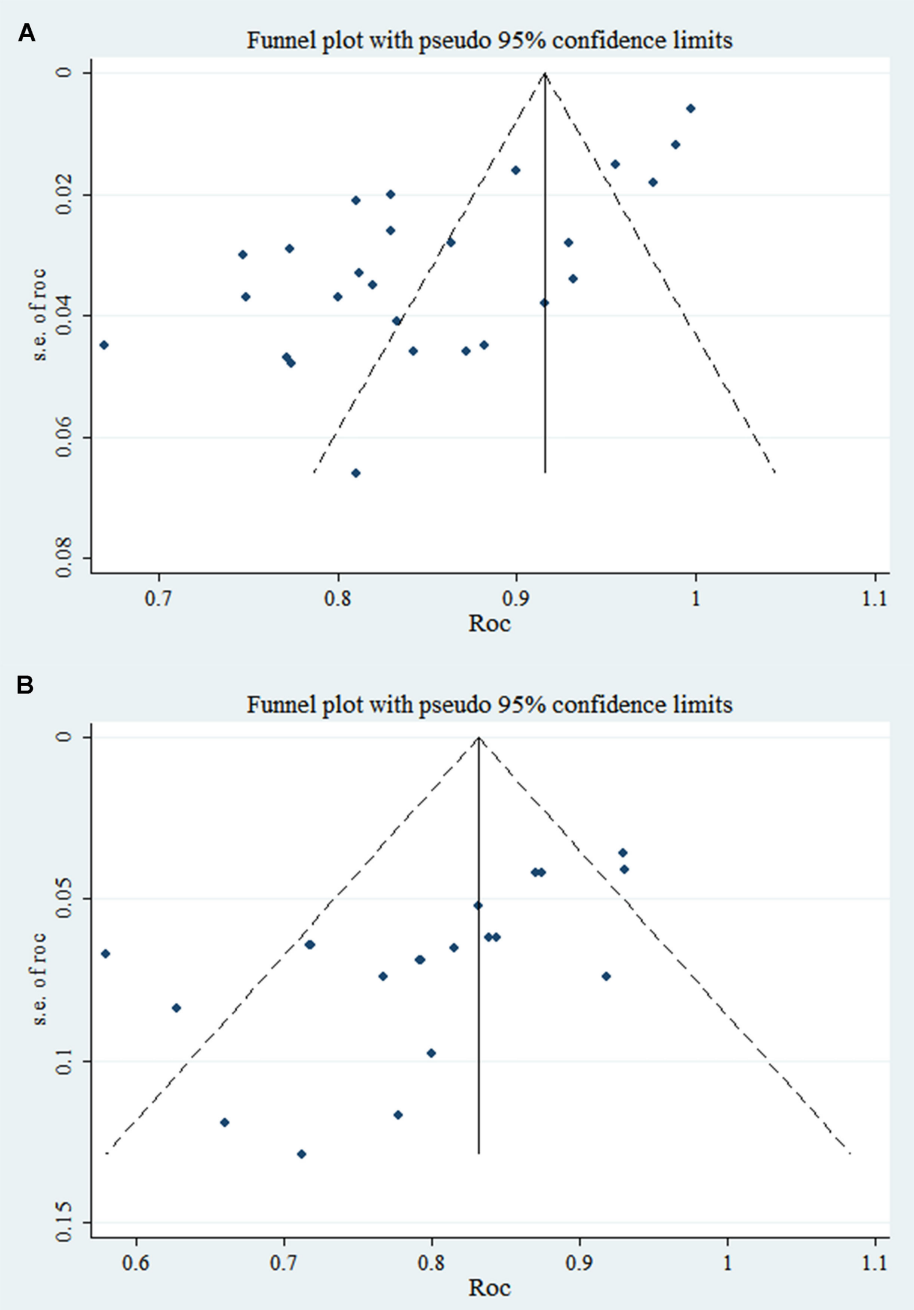
C-index funnel plot of TDs (note: **A** is training set; **B** is validation set).

In the training and validation sets, some studies appear to have publication bias (PB), as indicated by Egger’s test with p < 0.001. The c-index funnel plot is shown in **Figure 4**.

#### 3.4.2 Perineural invasion

##### 3.4.2.1 Synthesized results

**Figure 5:**
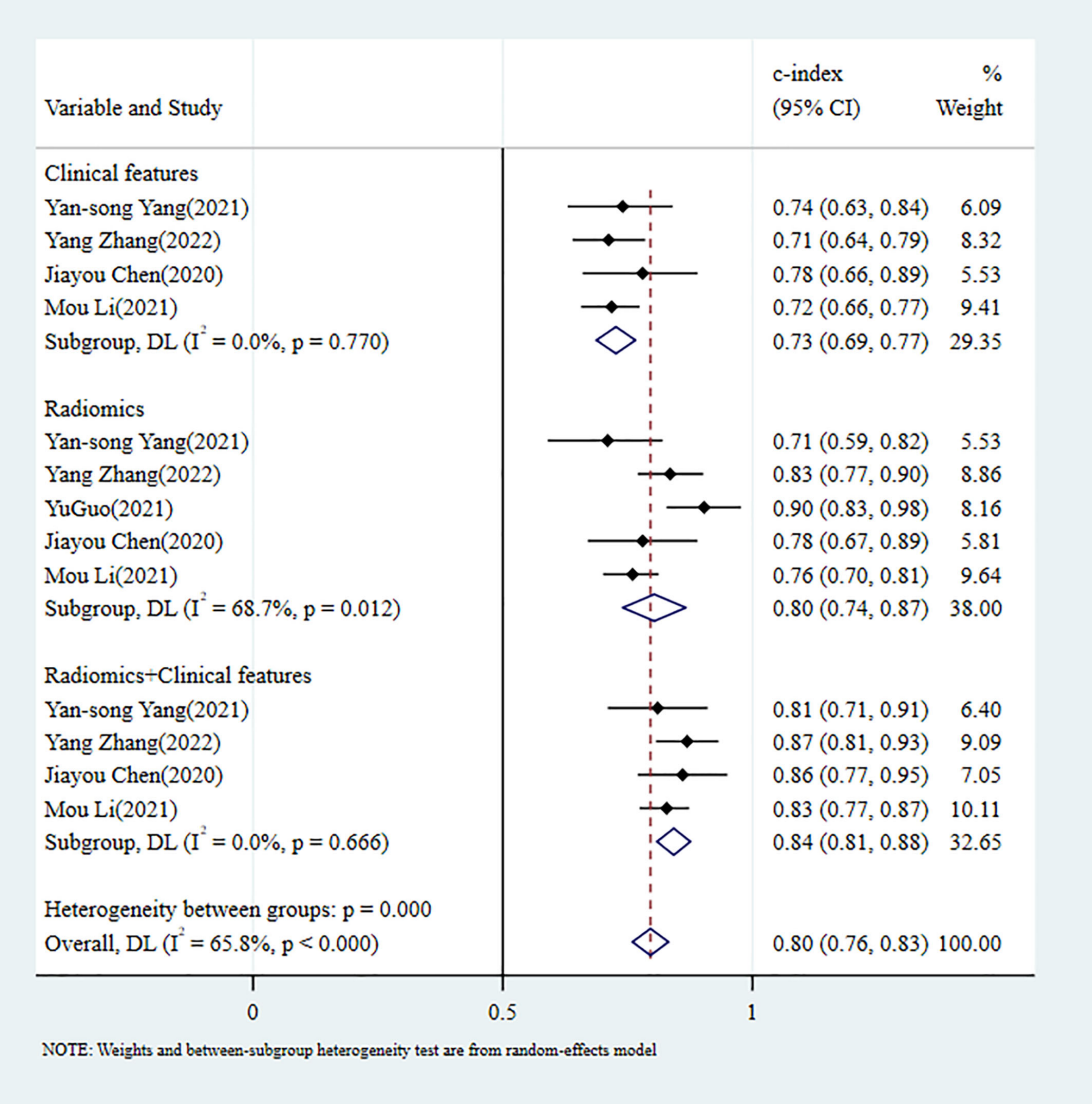
C-index meta-analysis forest diagram for diagnosing PNI using machine learning models in the training set.

In the training set, 5 MLMs were constructed based on RFs alone. The c-index, sensitivity, and specificity, as summarized using a random-effects model, were 0.80 (95% CI: 0.74 - 0.87), 0.69 (95% CI: 0.58 - 0.78), and 0.78 (95% CI: 0.66 - 0.87), respectively. Four MLMs were constructed based solely on CFs; a random-effects model was used, and the pooled c-index, sensitivity, and specificity were 0.73 (95% CI: 0.69 - 0.77), 0.63 (95% CI: 0.56 - 0.69), and 0.72 (95% CI: 0.68 - 0.76), respectively. Four MLMs were developed based on RFs combined with interpretable CFs; a random-effects model was used, and the pooled c-index, sensitivity, and specificity were 0.84 (95% CI: 0.81 - 0.88), 0.65 (95% CI: 0.57 - 0.72), and 0.89 (95% CI: 0.85 - 0.93), respectively. MLMs based on RFs alone or on the combination of RFs and CFs outperformed MLMs based on CFs alone. The forest maps of c-index and the sensitivity and specificity obtained from different modeling variables are shown in **Figure 5** and **Supplementary Figures S7**-**S9**.

**Figure 6:**
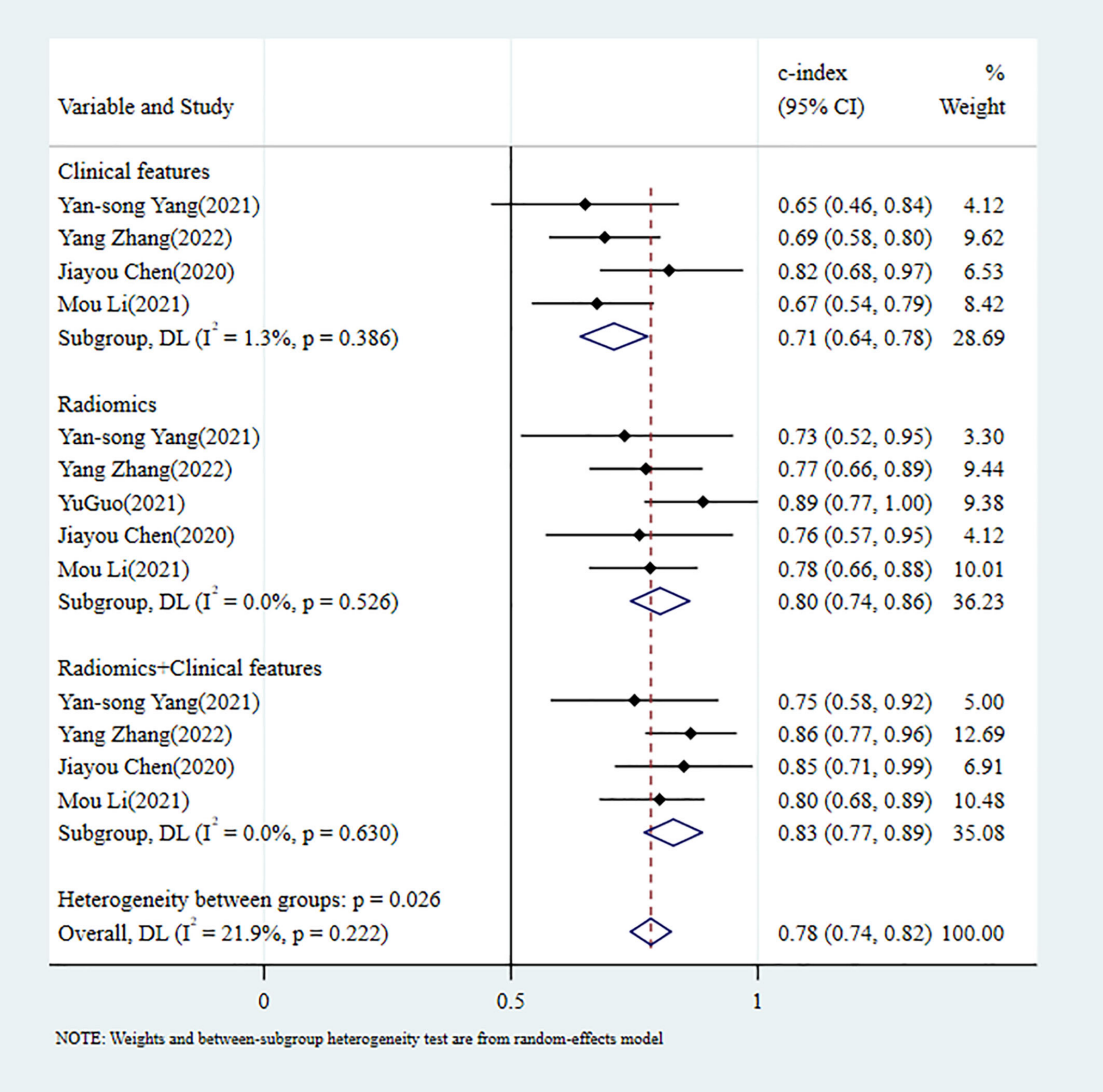
C-index meta-analysis forest diagram for diagnosing PNI using machine learning models in the validation set.

In the validation set, 5 MLMs were constructed based on RFs alone. The c-index, sensitivity, and specificity, as summarized using a random-effects model, were 0.80 (95% CI: 0.74 - 0.86), 0.64 (95% CI: 0.44 - 0.80), and 0.79 (95% CI: 0.68 - 0.87), respectively. Four MLMs were constructed based solely on CFs; a random-effects model was used, and the pooled c-index, sensitivity, and specificity were 0.71 (95% CI: 0.64 - 0.78), 0.63 (95% CI: 0.51 - 0.73), and 0.76 (95% CI: 0.69 - 0.82), respectively. Four MLMs were developed based on RFs combined with interpretable CFs; a random-effects model was used, and the pooled c-index, sensitivity, and specificity were 0.83 (95% CI: 0.77 - 0.89), 0.60 (95% CI: 0.48 - 0.71), and 0.90 (95% CI: 0.84 - 0.94), respectively. MLMs based on RFs alone or on the combination of RFs and CFs outperformed MLMs based on CFs alone. The forest maps of c-index and the sensitivity and specificity obtained from different modeling variables are shown in **Figure 6** and **Supplementary Figures S10**–**S12**.

##### 3.4.2.2 Reporting biases

**Figure 7:**
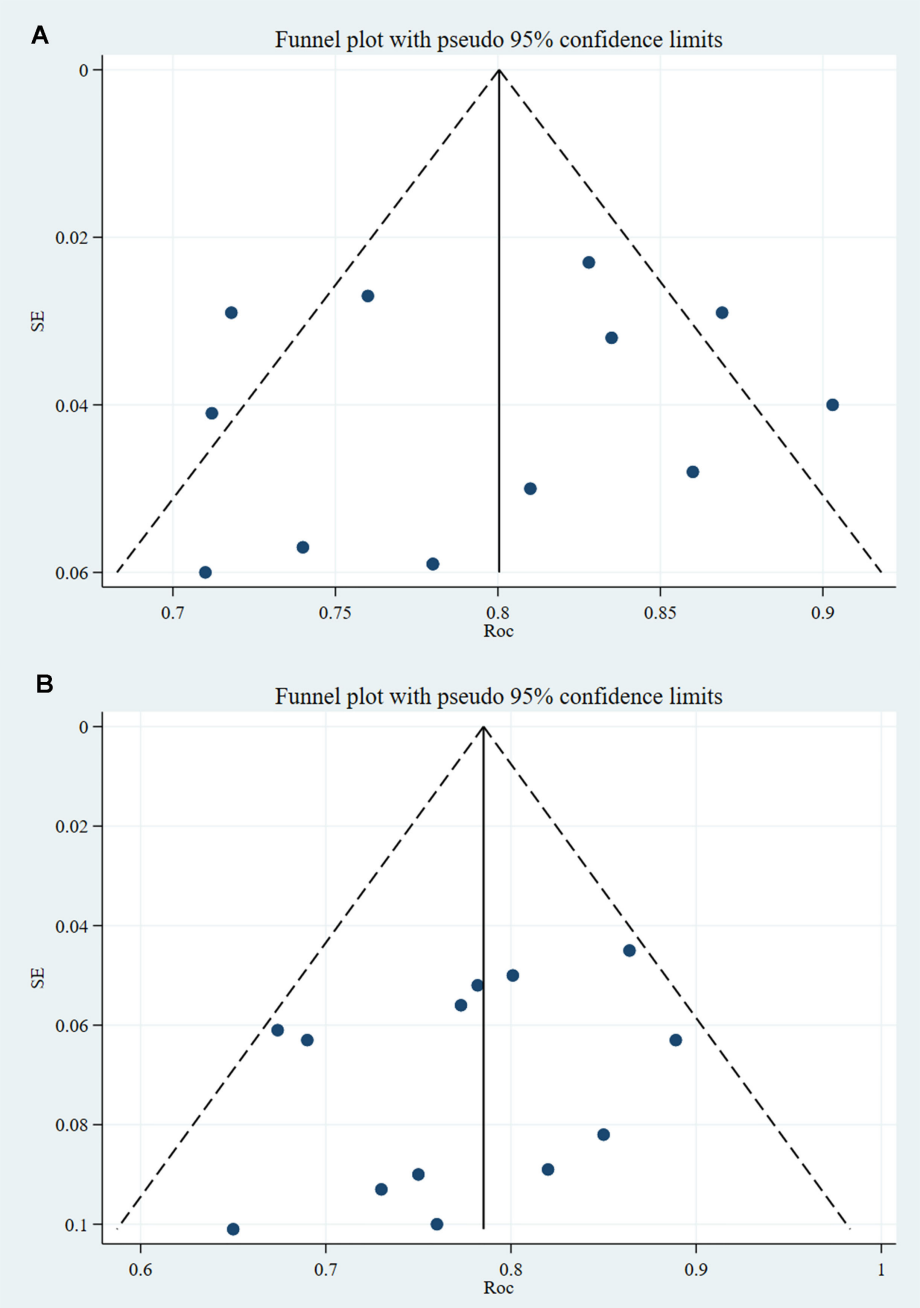
C-index funnel plot of PNI (note: **A** is training set; **B** is validation set).

Both the training and validation sets did not include any PBs in the studies analyzed, and the p-values from Egger’s test were 0.593 and 0.234, respectively. The c-index funnel plot is shown in **Figure 7**.

## 4 Discussion

### 4.1 Summary of main findings

The study on TDs revealed that MLMs based on both RFs and CFs demonstrated significantly better diagnostic performance than models constructed solely on CFs or RFs. The c-index was 0.87 (95% CI: 0.83 - 0.91) and the sensitivity was 0.91 (95% CI: 0.72 - 0.99). However, the specificity was relatively low at 0.65 (95% CI: 0.53 - 0.76). The study on PNI demonstrated that MLMs based on both RFs and CFs had significantly better diagnostic performance than models constructed solely on CFs or RFs. The c-index was 0.83 (95% CI: 0.77 - 0.89), and the specificity was 0.90 (95% CI: 0.84 - 0.94). However, the sensitivity of all three models was inadequate.

### 4.2 Comparison with previous reviews

Some investigators are exploring non-invasive methods to identify TDs and PNI that have been associated with adverse outcomes in previous studie**s** (3, 31). Therefore, some investigators have examined the efficacy of conventional imaging and clinical factors in identifying TDs and PNI in RC. In a retrospective analysis of MRI images of 130 patients, Lv et al. (32) constructed a nomogram model using MRI-detected TDs and metastatic lymph nodes for predicting TDs. The results revealed that patients diagnosed with TDs and metastatic lymph nodes through MRI before surgery had a 52% chance of ultimately being diagnosed with TDs through pathology. The nomogram model demonstrated an AUC of 0.814 (0.720 - 0.908), with a sensitivity of 73.9% and a specificity of 79.4%. Chen et al. conducted a meta-analysis on PNI using the included studies and developed a model for diagnosing PNI based on CFs. The resulting AUC was 0.768, with a sensitivity of 0.947 and a specificity of 0.358 (33). This study revealed that models utilizing both RFs and CFs to extract deep information from images outperformed conventional imaging or models utilizing CFs alone, regardless of TDs or PNI. These findings suggest that radiomics has enormous potential in the field.

Although current studies have shown a significant correlation between RFs and specific tumor biomaterials (34), a single RF lacks interpretability (35). Compensating for the shortcomings of a single RF, CFs can provide deeper information in images. Chen et al. (33) revealed through a meta-analysis that T staging, vascular invasion, and degree of differentiation are closely related to PNI, which is a pathological manifestation of a disease. Jhuang et al. (36) discovered that the state of lymphatic vessel invasion is closely related to TDs. These studies suggest that certain CFs are closely associated with PNI and TDs. Therefore, MLMs that combine interpretable CFs with more informative RFs often have good diagnostic performance, which is consistent with the findings of the present study.

When selecting MLMs, it is important to strike a balance between interpretability and accuracy. Models that are highly interpretable, such as logistic regression and decision trees, tend to have lower accuracy. Conversely, models with higher accuracy, such as neural networks and deep learning, often lack interpretability (37). In the studies analyzed in this systematic review and meta-analysis, the MLMs were mainly based on logistic regression. Only two studies employed deep learning models, and the AUC of the RF- and CF-based models was 0.83 and 0.88, respectively. The diagnostic performance of deep learning models has not been significantly improved compared to studies using LRMs. However, this conclusion is based on extremely limited evidence. Therefore, more studies are required to validate the clinical value of deep learning models.

RQS is an objective tool for evaluating the quality of radiomics studies (14). To ensure accuracy, scoring results in this study were obtained independently by two experienced investigators (38). Most of the studies were single-center studies without external validation, resulting in relatively low RQS scores. This is consistent with the results of previous studies (39). Improving the quality of radiomics studies is heavily reliant on conducting multicenter studies. However, while focusing on enhancing study quality, it is important to note that the applicability of RQS system still needs improvement. The system suggests repeated measurements of patients in a short period of time, which contradicts ethics and clinical practice. Additionally, the RQS system aims to promote the public disclosure of code and data by investigators. However, this is currently challenging to achieve. At present, RQS mainly focuses on radiomics studies using traditional machine learning. Its scope encompasses the evaluation of image sources, image segmentation, texture extraction, model construction and validation. For radiomics studies using deep learning, texture extraction is usually directly integrated into the modeling process, which often leads to low RQS (39). Therefore, it is necessary to continuously improve the RQS system in the future.

### 4.3 Strengths and limitations

This is the first systematic review and meta-analysis of machine learning performance in assessing PNI and TDs in RC. It provides evidence-based support for the radiomic diagnosis of TDs and PNI in RC for the first time. In the process of analysis, the present study summarized the clinical feature model, the radiomics model, and the combination model, and highlighted the advantages of the radiomics model and the combination model. In addition, the results of the training and validation sets from the included studies were analyzed to demonstrate the suitability of each model. However, this study has its limitations. Most of the included studies were conducted in China, and most of them were single-center studies, which greatly limits the generalizability of the results. Furthermore, although a systematic search was conducted, the limited number of studies on radiomics for diagnosing TDs and nerve invasion in RC restricted the number of studies included in this study, which in turn limited the interpretation of results. Moreover, additional subgroup analyses, such as those involving different imaging devices and study models, were not possible because of the limited number of studies. Therefore, future studies need to focus on the generalizability of models across multiple centers and countries to boost its overall applicability. At the same time, deep learning models are very scarce and need further development and validation.

## 5 Conclusions

As a result of ongoing advances in medical studies, the treatment strategy for RC is being continuously optimized. In the individualized treatment for RC, surgical resection is not the only preferred method. Imaging-based preoperative examinations can inform different treatment decisions for clinical practice. The diagnostic model, constructed on both RFs and CFs, demonstrates good diagnostic performance for PNI and TDs in RC. It is expected to become a non-invasive detection method. Further development and improvement of radiomic models may provide valuable insights in clinical practice, enabling accurate diagnosis of PNI and TDs in RC.

## Data Availability

The original contributions presented in the study are included in the article/**Supplementary Material**. Further inquiries can be directed to the corresponding author.

## Supplementary material

Supplementary Figure 1 Sensitivity and specificity of the training set based on radiomics feature models for diagnosing TDs.

Supplementary Figure 2 Sensitivity and specificity of the training set based on clinical feature models for diagnosing TDs.

Supplementary Figure 3 Sensitivity and specificity of the training set based on both radiomics feature models and clinical feature models for diagnosing TDs.

Supplementary Figure 4 Sensitivity and specificity of the validation set based on radiomics feature models for diagnosing TDs.

Supplementary Figure 5 Sensitivity and specificity of the validation set based on clinical feature models for diagnosing TDs.

Supplementary Figure 6 Sensitivity and specificity of the validation set based on both radiomics feature models and clinical feature models for diagnosing TDs.

Supplementary Figure 7 Sensitivity and specificity of the training set based on radiomics feature models for diagnosing PNI.

Supplementary Figure 8 Sensitivity and specificity of the training set based on clinical feature models for diagnosing PNI.

Supplementary Figure 9 Sensitivity and specificity of the training set based on both radiomics feature models and clinical feature models for diagnosing PNI.

Supplementary Figure 10 Sensitivity and specificity of the validation set based on radiomics feature models for diagnosing PNI.

Supplementary Figure 11 Sensitivity and specificity of the validation set based on clinical feature models for diagnosing PNI.

Supplementary Figure 12 Sensitivity and specificity of the validation set based on both radiomics feature models and clinical feature models for diagnosing PNI.
