# Supplementary figures and images for: The accuracy of radiomics in diagnosing tumor deposits and perineural invasion in rectal cancer: a systematic review and meta-analysis
Source: Front Oncol. 2025 Jan 8;14:1425665. doi: 10.3389/fonc.2024.1425665 (PMC11750663; doi:10.3389/fonc.2024.1425665)

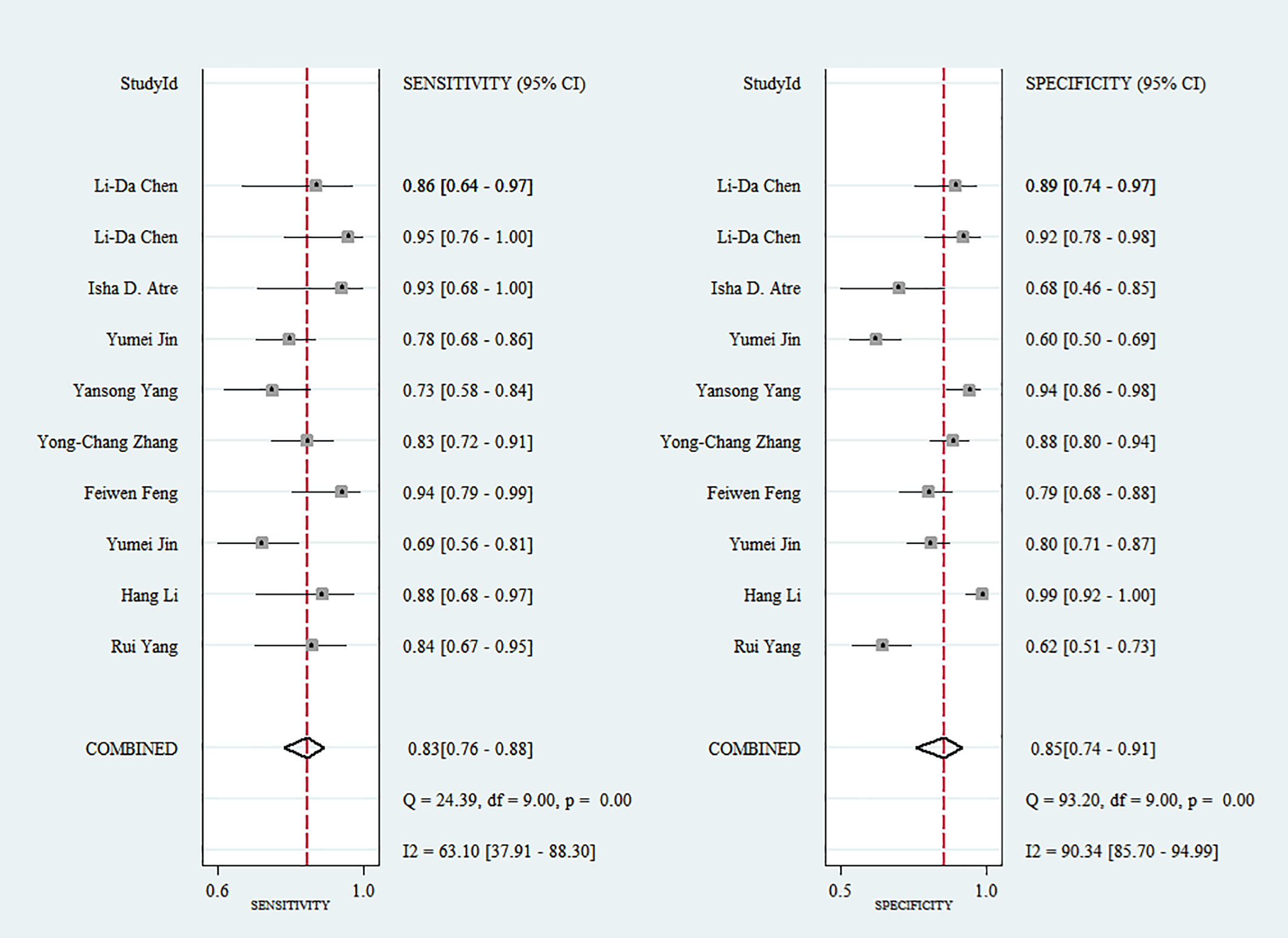

Supplement: Supplementary Figure 1 — Sensitivity and specificity of the training set based on radiomics feature models for diagnosing TDs. [file Image1.tif]

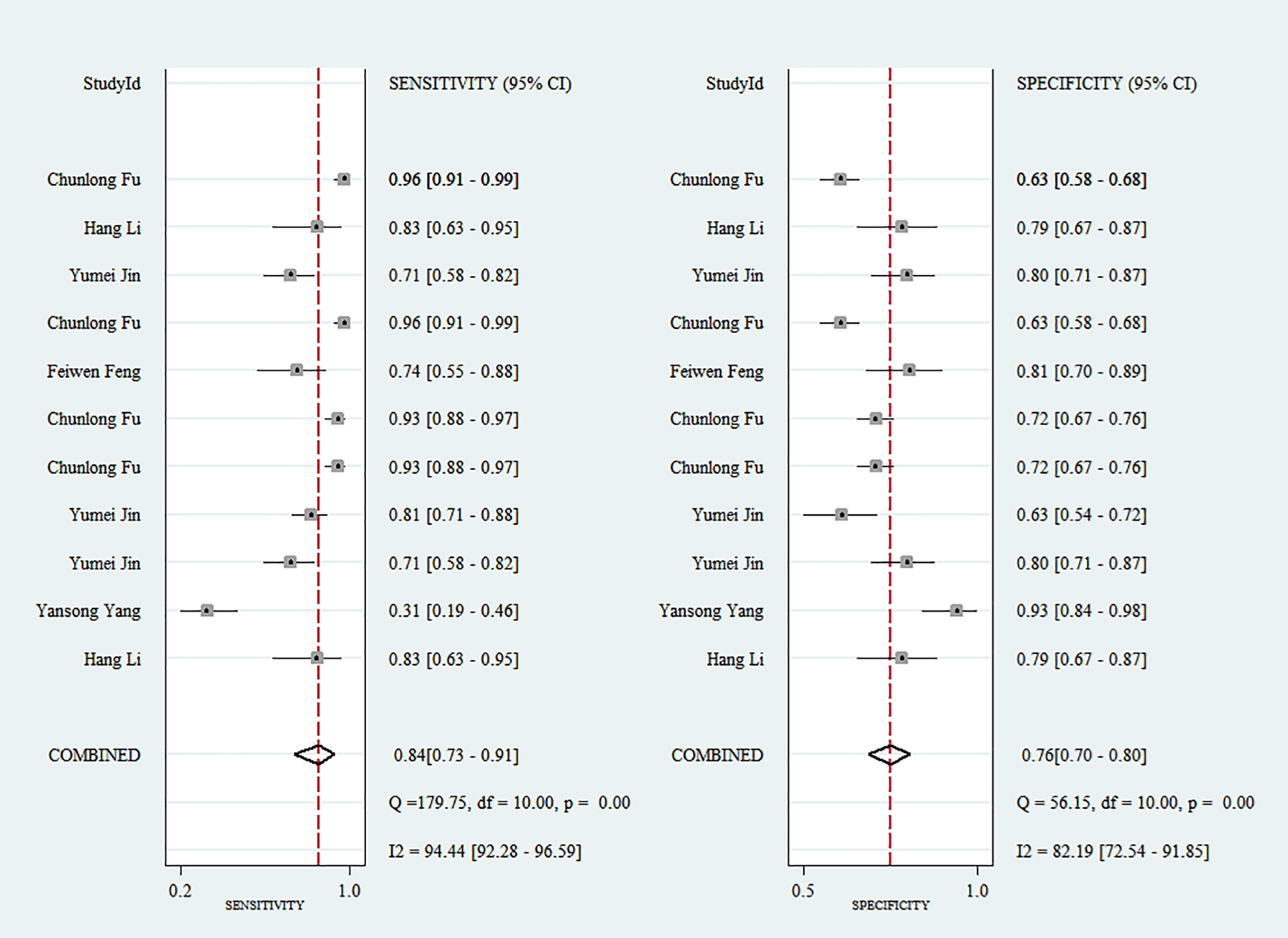

Supplement: Supplementary Figure 2 — Sensitivity and specificity of the training set based on clinical feature models for diagnosing TDs. [file Image2.tif]

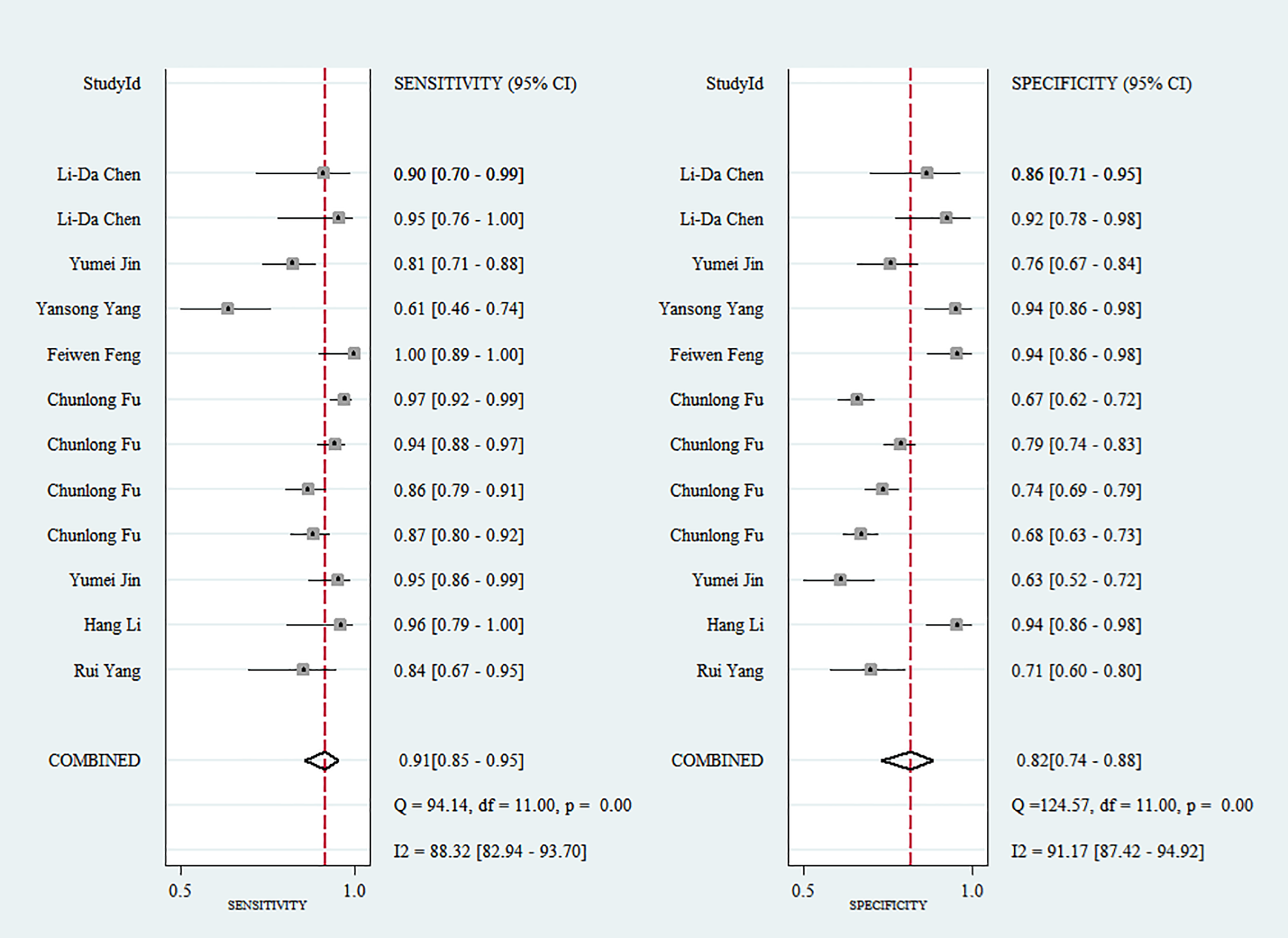

Supplement: Supplementary Figure 3 — Sensitivity and specificity of the training set based on both radiomics feature models and clinical feature models for diagnosing TDs. [file Image3.tif]

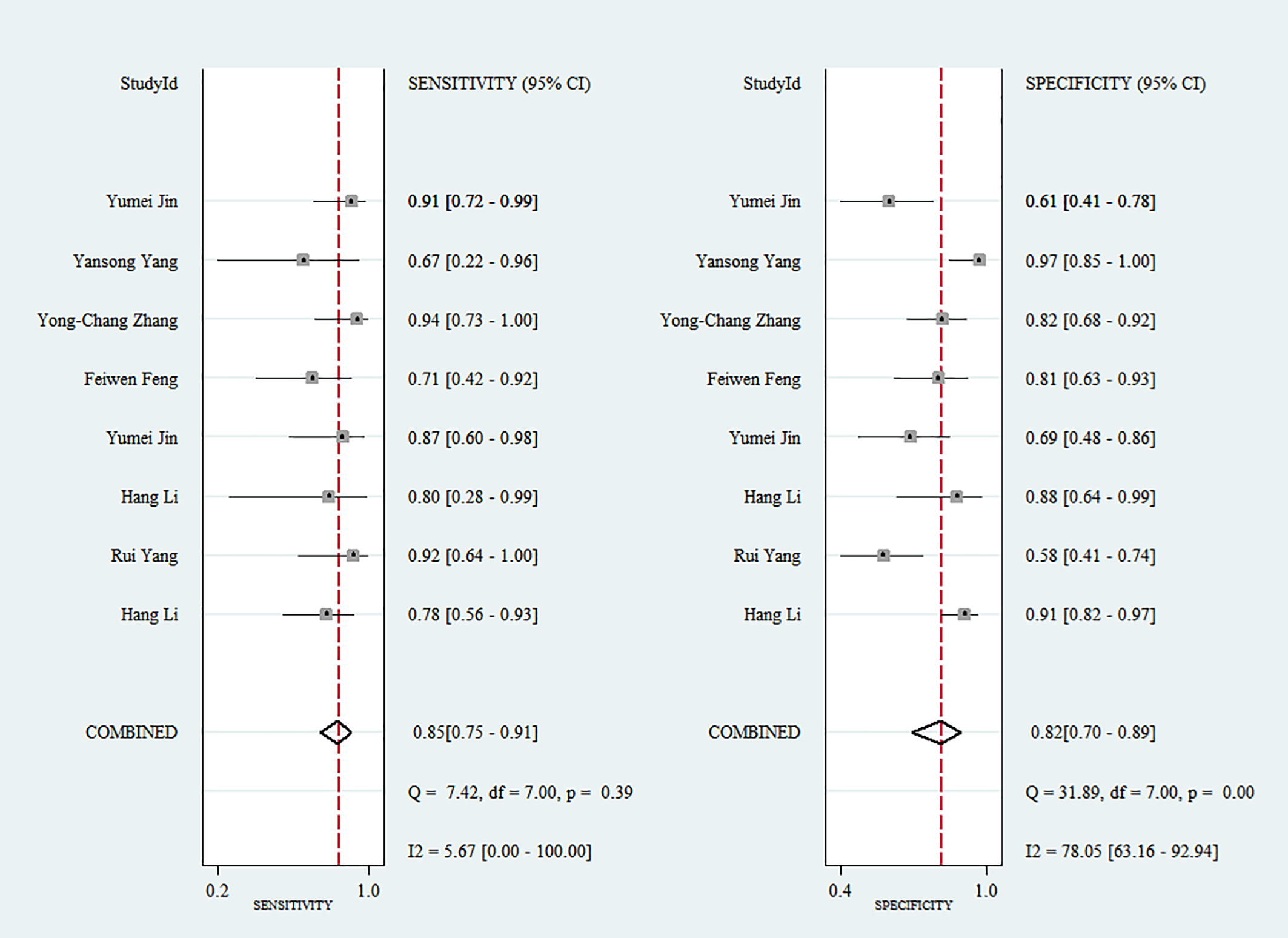

Supplement: Supplementary Figure 4 — Sensitivity and specificity of the validation set based on radiomics feature models for diagnosing TDs. [file Image4.tif]

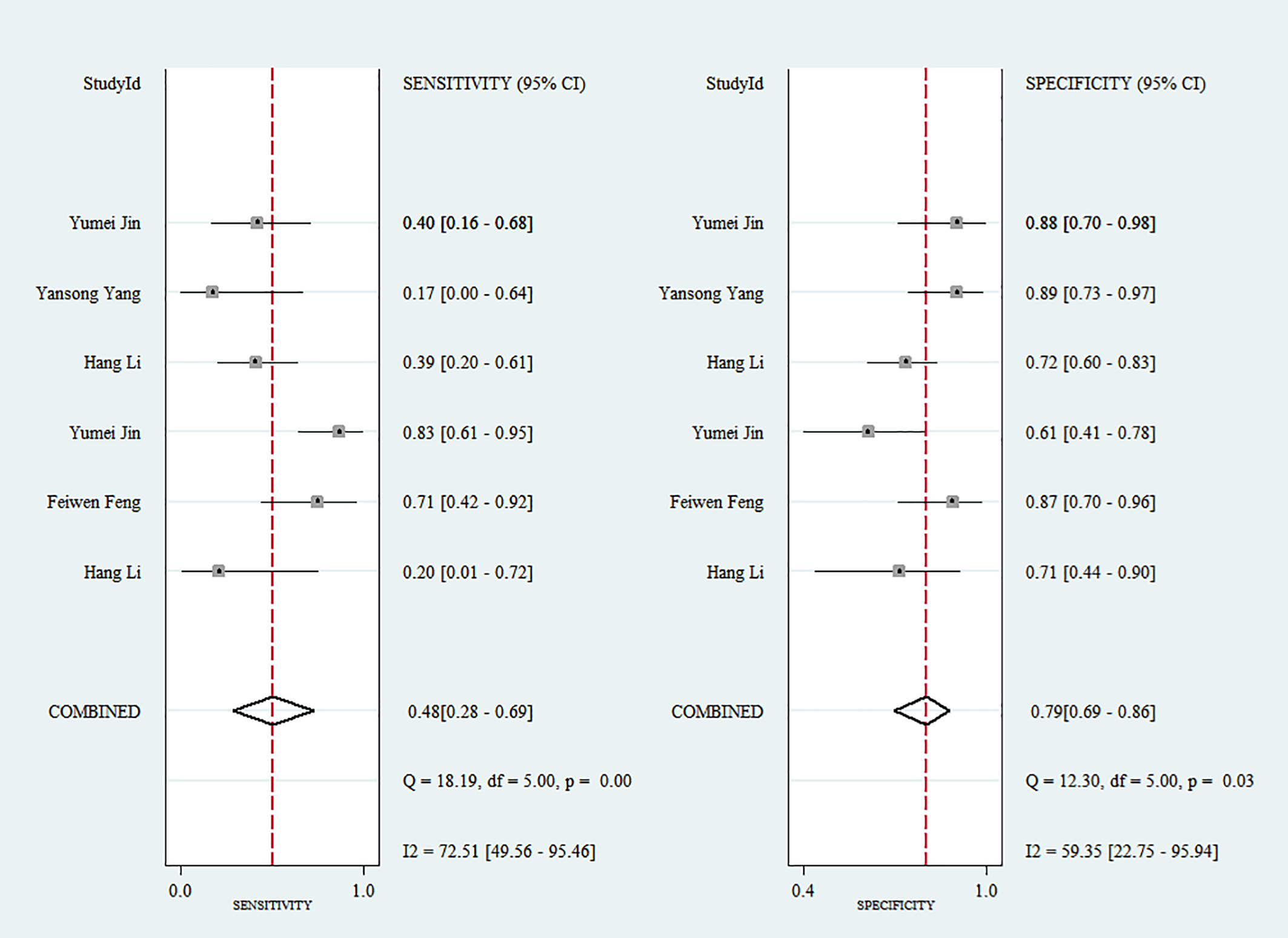

Supplement: Supplementary Figure 5 — Sensitivity and specificity of the validation set based on clinical feature models for diagnosing TDs. [file Image5.tif]

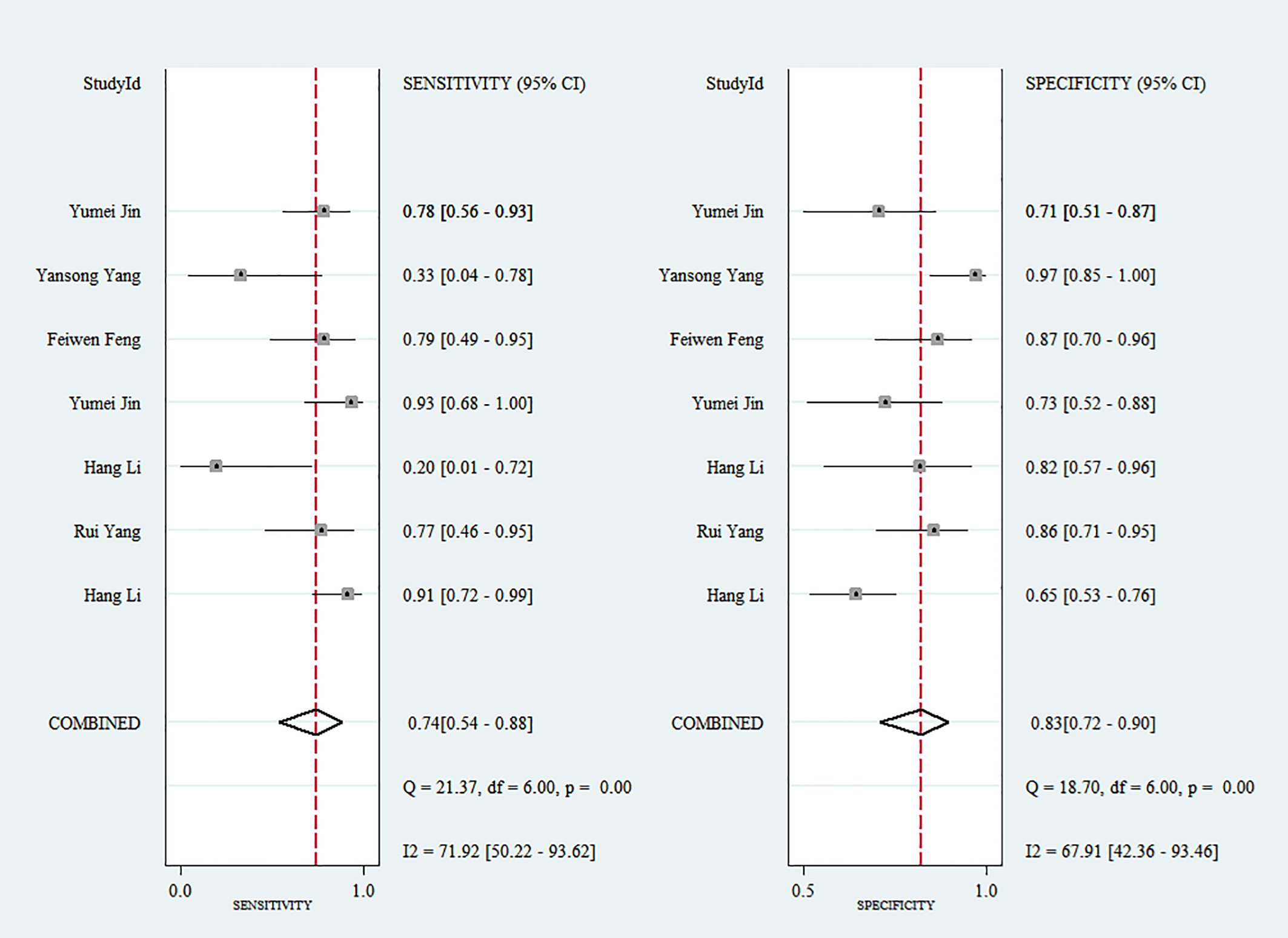

Supplement: Supplementary Figure 6 — Sensitivity and specificity of the validation set based on both radiomics feature models and clinical feature models for diagnosing TDs. [file Image6.tif]

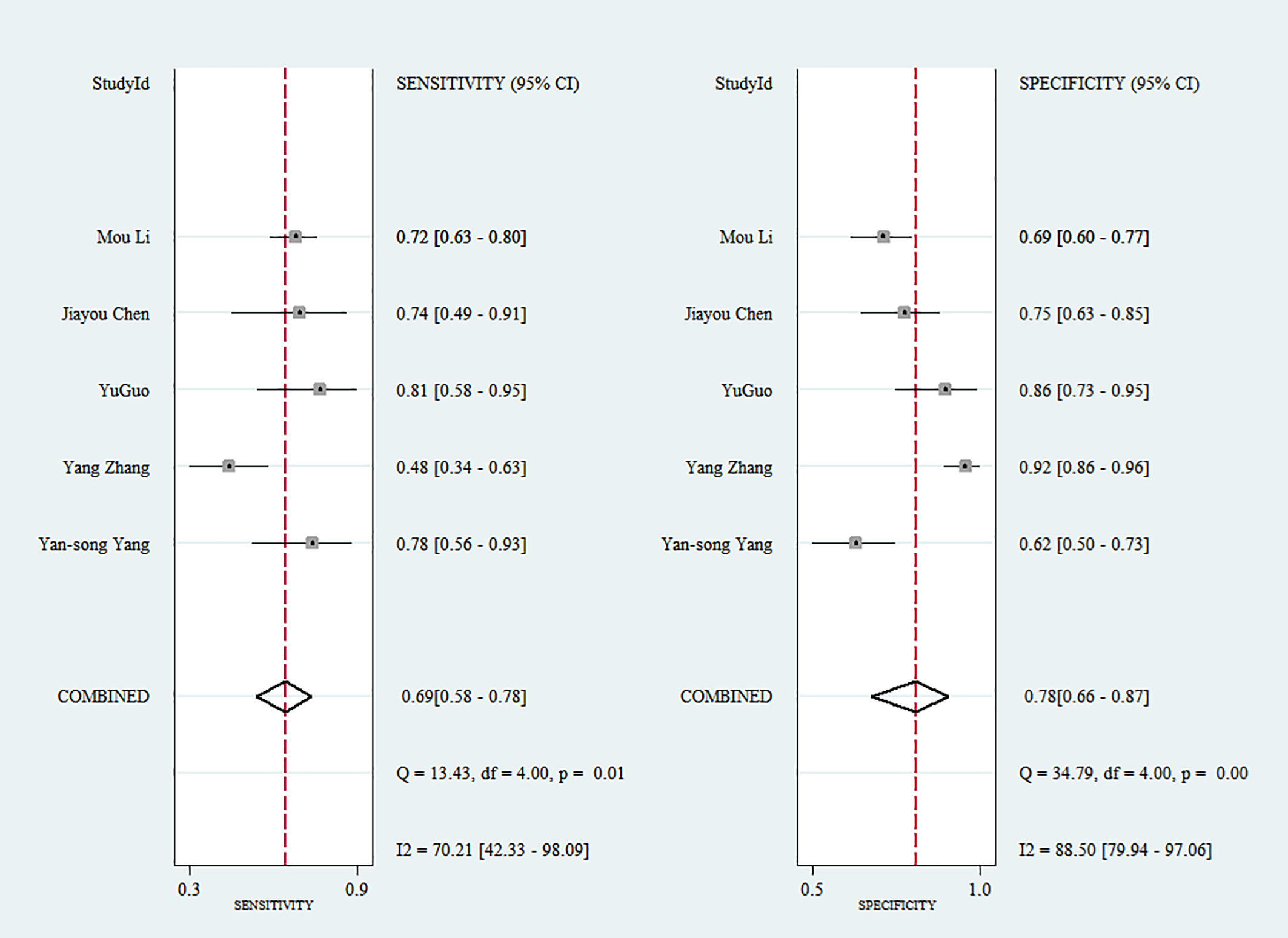

Supplement: Supplementary Figure 7 — Sensitivity and specificity of the training set based on radiomics feature models for diagnosing PNI. [file Image7.tif]

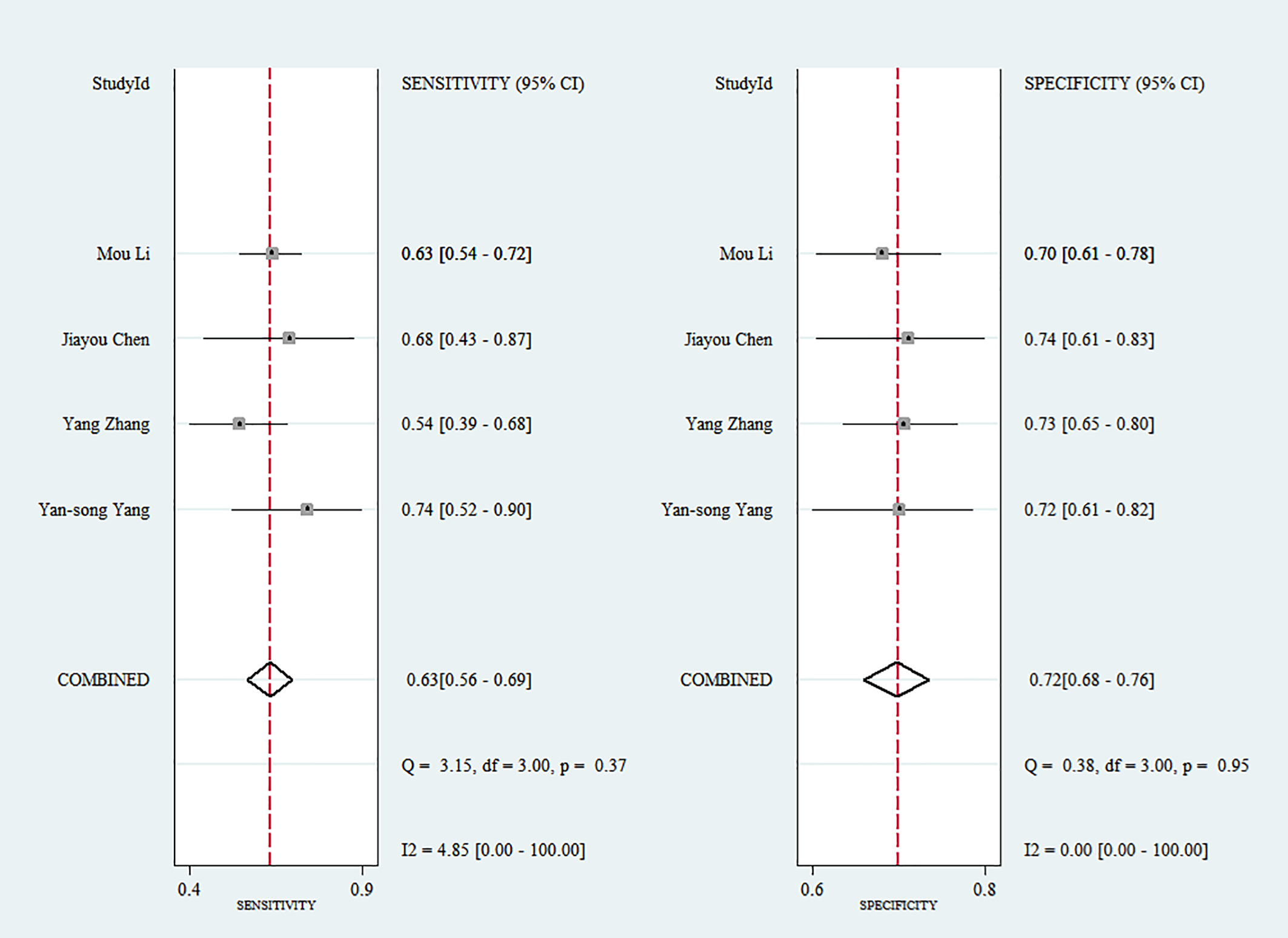

Supplement: Supplementary Figure 8 — Sensitivity and specificity of the training set based on clinical feature models for diagnosing PNI. [file Image8.tif]

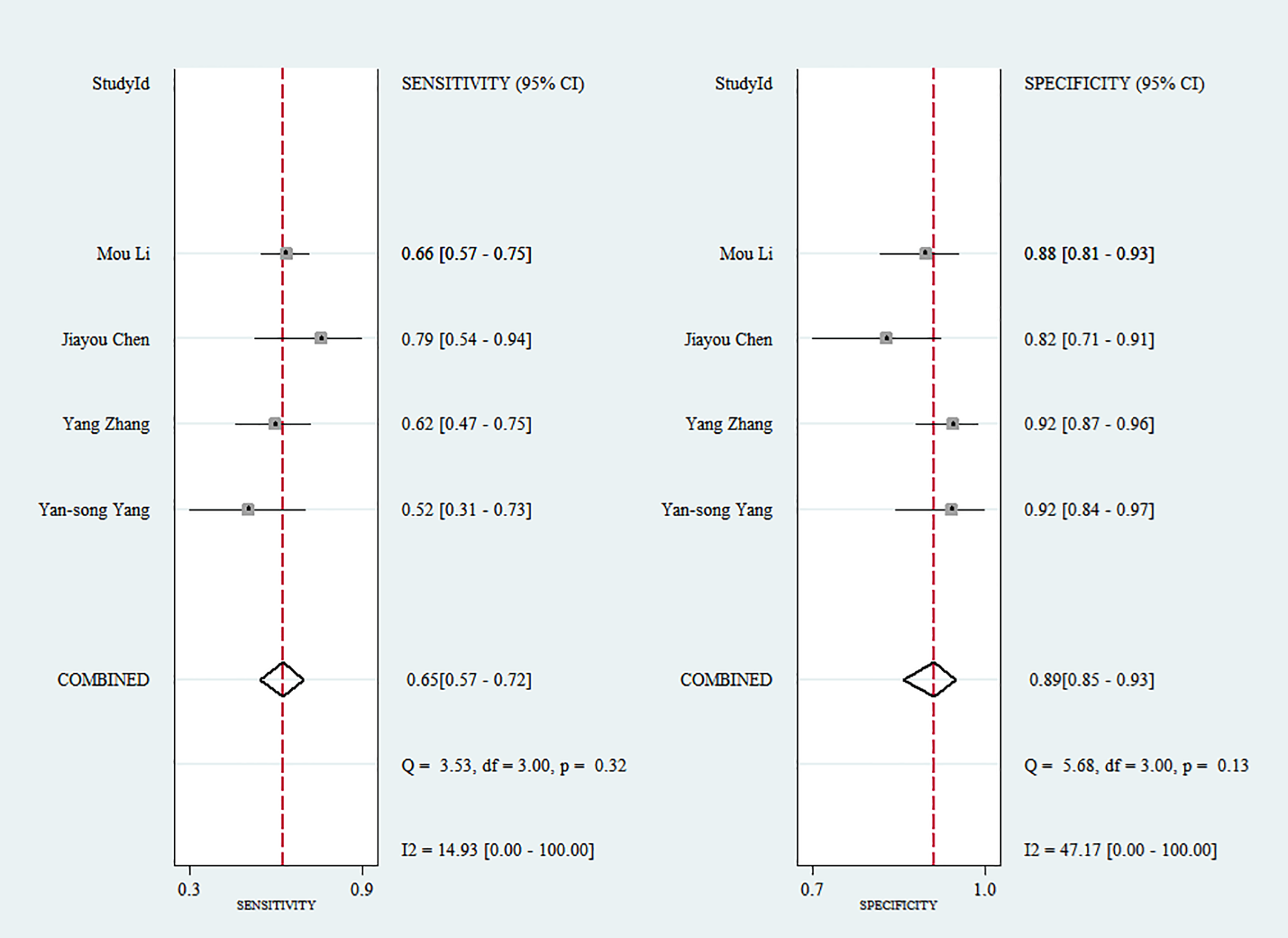

Supplement: Supplementary Figure 9 — Sensitivity and specificity of the training set based on both radiomics feature models and clinical feature models for diagnosing PNI. [file Image9.tif]

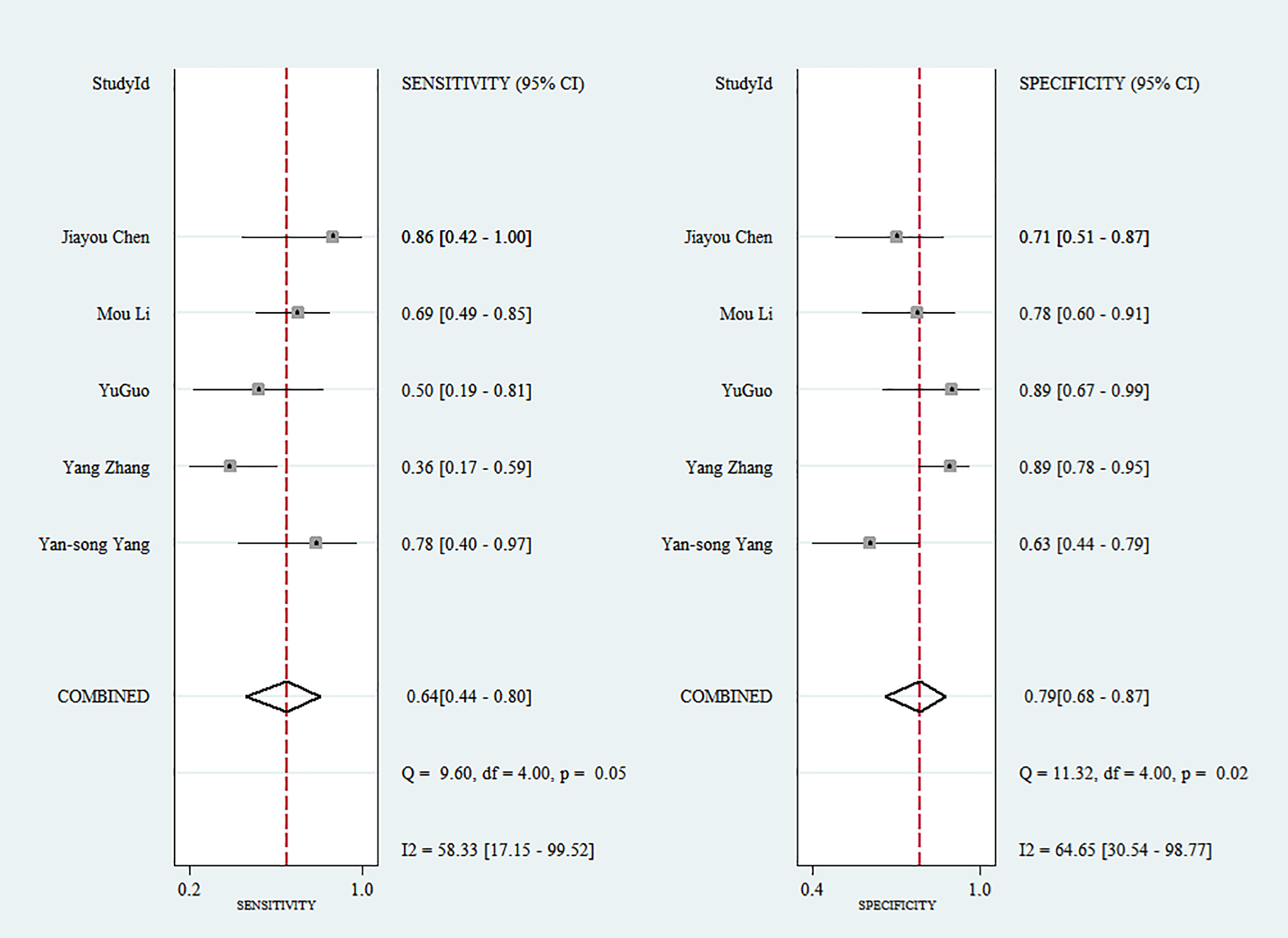

Supplement: Supplementary Figure 10 — Sensitivity and specificity of the validation set based on radiomics feature models for diagnosing PNI. [file Image10.tif]

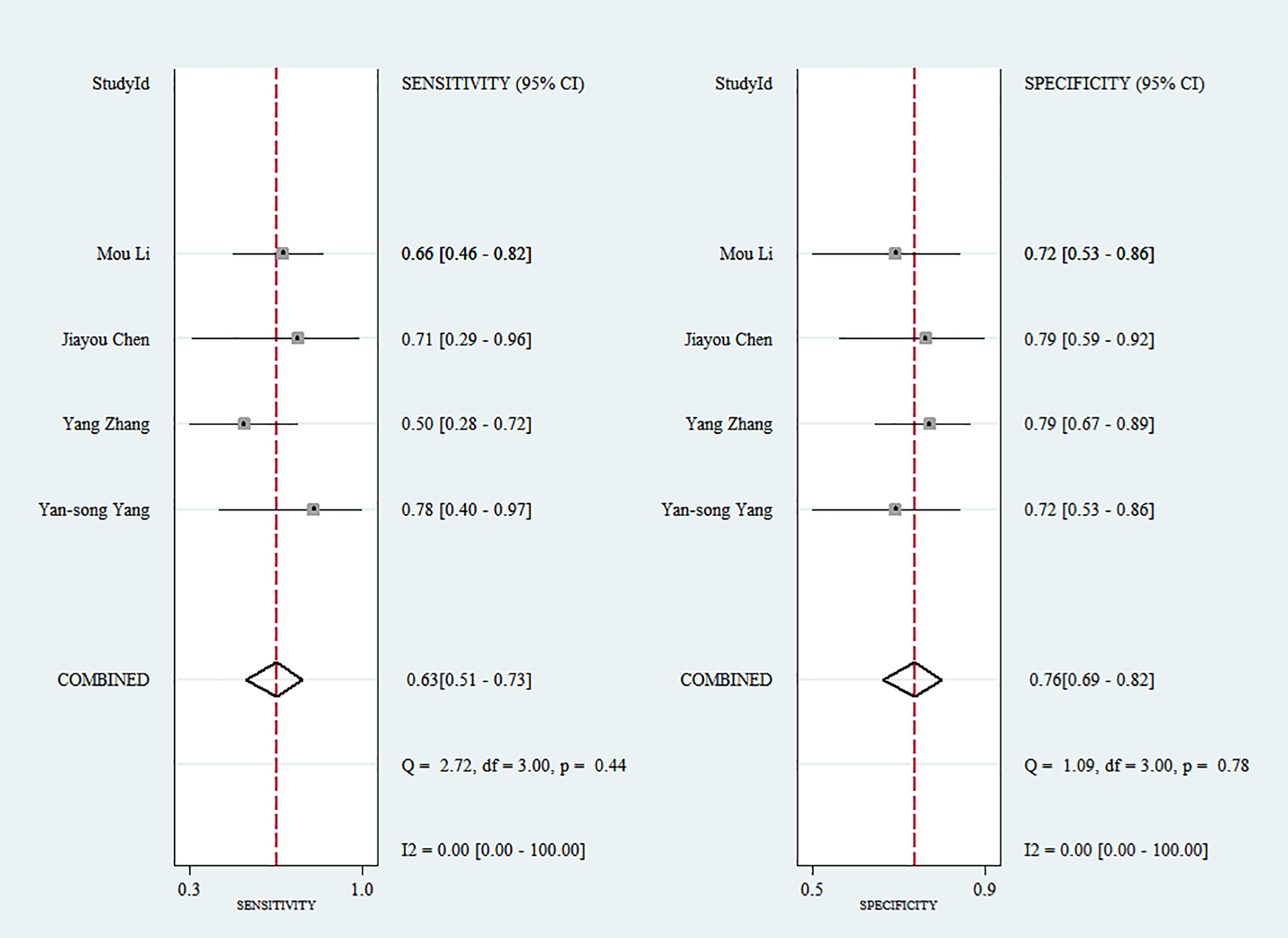

Supplement: Supplementary Figure 11 — Sensitivity and specificity of the validation set based on clinical feature models for diagnosing PNI. [file Image11.tif]

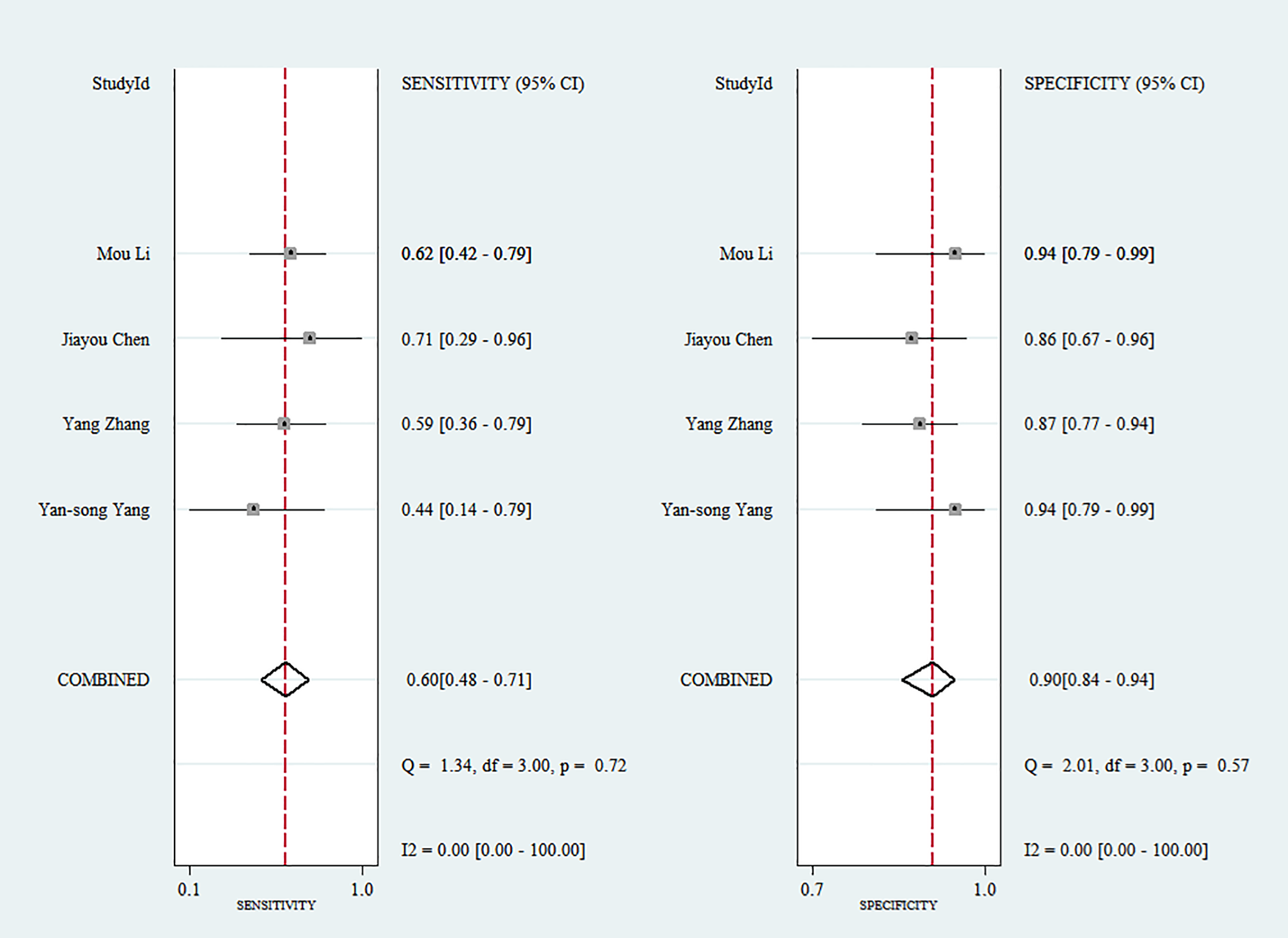

Supplement: Supplementary Figure 12 — Sensitivity and specificity of the validation set based on both radiomics feature models and clinical feature models for diagnosing PNI. [file Image12.tif]
