# Supplementary material for: The accuracy of radiomics in diagnosing tumor deposits and perineural invasion in rectal cancer: a systematic review and meta-analysis
Source: Front Oncol. 2025 Jan 8;14:1425665. doi: 10.3389/fonc.2024.1425665 (PMC11750663; doi:10.3389/fonc.2024.1425665)
Supplement: Supplementary file 13 [file Table1.docx]

# Table S1 Literature search strategy

**1.Pubmed**

| Search number | Query | Results |
| --- | --- | --- |
| #1 | "Rectal Neoplasms"[Title/Abstract] OR "Cancer of Rectum"[Title/Abstract] OR "Cancer of the Rectum"[Title/Abstract] OR "neoplasm of the rectum"[Title/Abstract] OR "neoplasma recti"[Title/Abstract] OR "pararectal tumor"[Title/Abstract] OR "pararectal tumour"[Title/Abstract] OR "Rectal Cancer*"[Title/Abstract] OR "rectal mass"[Title/Abstract] OR "rectal neoplasia"[Title/Abstract] OR "rectal neoplasm"[Title/Abstract] OR "rectal tumor*"[Title/Abstract] OR "rectal tumour"[Title/Abstract] OR "Rectum Cancer*"[Title/Abstract] OR "rectum mass"[Title/Abstract] OR "rectum neoplasia"[Title/Abstract] OR "rectum neoplasm*"[Title/Abstract] OR "rectum tumor"[Title/Abstract] OR "rectum tumour"[Title/Abstract] OR "retrorectal tumor"[Title/Abstract] OR "retrorectal tumour"[Title/Abstract] OR "tumor of the rectum"[Title/Abstract] OR "tumor recti"[Title/Abstract] OR "tumour of the rectum"[Title/Abstract] OR "tumour recti"[Title/Abstract] | 36719 |
| #2 | Rectal Neoplasms[MeSH Terms] | 54892 |
| #3 | "Radiomics"[Title/Abstract] OR "radiomic"[Title/Abstract] OR "radiogenomic"[Title/Abstract] OR "radiomics-based"[Title/Abstract] OR "Texture"[Title/Abstract] OR "Transfer Learning"[Title/Abstract] OR "Deep learning"[Title/Abstract] OR "Ensemble Learning"[Title/Abstract] OR "artificial intelligence"[Title/Abstract] OR "random forest"[Title/Abstract] OR "neural network"[Title/Abstract] OR "neural networks"[Title/Abstract] OR "K-Nearest Neighbor"[Title/Abstract] OR "CNN"[Title/Abstract] OR "AlexNet"[Title/Abstract] OR "VGGNet"[Title/Abstract] OR "ResNet"[Title/Abstract] OR "GoogLeNet"[Title/Abstract] OR "Support vector machine"[Title/Abstract] OR "SVM"[Title/Abstract] OR "Gradient Boosting Machine"[Title/Abstract] OR "Nomogram"[Title/Abstract] OR "XGBoost"[Title/Abstract] OR "Adaboost"[Title/Abstract] OR "Decision tree"[Title/Abstract] OR "ResNet-50"[Title/Abstract] OR "ResNet"[Title/Abstract] OR "Naive Bayesian"[Title/Abstract] OR "Multilayer perceptron"[Title/Abstract] OR "Bayesian network"[Title/Abstract] | 284744 |
| #4 | Machine Learning[MeSH Terms] | 62310 |
| #5 | (#1 OR #2) AND (#3 OR #4) | 842 |

**2.Cochrane**

| Search number | Query | Results |
| --- | --- | --- |
| #1 | ('Rectal Neoplasms' OR 'Cancer of Rectum' OR 'Cancer of the Rectum' OR 'neoplasm of the rectum' OR 'neoplasma recti' OR 'pararectal tumor' OR 'pararectal tumour' OR 'Rectal Cancer*' OR 'rectal mass' OR 'rectal neoplasia' OR 'rectal neoplasm' OR 'rectal tumor*' OR 'rectal tumour' OR 'Rectum Cancer*' OR 'rectum mass' OR 'rectum neoplasia' OR 'rectum neoplasm*' OR 'rectum tumor' OR 'rectum tumour' OR 'retrorectal tumor' OR 'retrorectal tumour' OR 'tumor of the rectum' OR 'tumor recti' OR 'tumour of the rectum' OR 'tumour recti' ):ti,ab,kw | 9395 |
| #2 | MeSH descriptor: [Rectal Neoplasms] explode all trees | 2506 |
| #3 | ('Radiomics' OR 'radiomic' OR 'radiogenomic' OR 'radiomics-based' OR 'Texture' OR 'Transfer Learning' OR 'Deep learning' OR 'Ensemble Learning' OR 'artificial intelligence' OR 'random forest' OR 'neural network' OR 'neural networks' OR 'K-Nearest Neighbor' OR 'CNN' OR 'AlexNet' OR 'VGGNet' OR 'ResNet' OR 'GoogLeNet' OR 'Support vector machine' OR 'SVM' OR 'Gradient Boosting Machine' OR 'Nomogram' OR 'XGBoost' OR 'Adaboost' OR 'Decision tree' OR 'ResNet-50' OR 'ResNet' OR 'Naive Bayesian' OR 'Multilayer perceptron' OR 'Bayesian network'):ti,ab,kw | 13274 |
| #4 | MeSH descriptor: [Machine Learning] explode all trees | 938 |
| #5 | (#1 OR #2) AND (#3 OR #4) | 147 |

**3.Embase**

| Search number | Query | Results |
| --- | --- | --- |
| #1 | 'rectal neoplasms':ti,ab,kw OR 'cancer of rectum':ti,ab,kw OR 'cancer of the rectum':ti,ab,kw OR 'neoplasm of the rectum':ti,ab,kw OR 'neoplasma recti':ti,ab,kw OR 'pararectal tumor':ti,ab,kw OR 'pararectal tumour':ti,ab,kw OR 'rectal cancer*':ti,ab,kw OR 'rectal mass':ti,ab,kw OR 'rectal neoplasia':ti,ab,kw OR 'rectal neoplasm':ti,ab,kw OR 'rectal tumor*':ti,ab,kw OR 'rectal tumour':ti,ab,kw OR 'rectum cancer*':ti,ab,kw OR 'rectum mass':ti,ab,kw OR 'rectum neoplasia':ti,ab,kw OR 'rectum neoplasm*':ti,ab,kw OR 'rectum tumor':ti,ab,kw OR 'rectum tumour':ti,ab,kw OR 'retrorectal tumor':ti,ab,kw OR 'retrorectal tumour':ti,ab,kw OR 'tumor of the rectum':ti,ab,kw OR 'tumor recti':ti,ab,kw OR 'tumour of the rectum':ti,ab,kw OR 'tumour recti':ti,ab,kw | 55191 |
| #2 | 'rectum tumor'/exp | 84781 |
| #3 | 'radiomics':ti,ab,kw OR 'radiomic':ti,ab,kw OR 'radiogenomic':ti,ab,kw OR 'radiomics-based':ti,ab,kw OR 'texture':ti,ab,kw OR 'transfer learning':ti,ab,kw OR 'deep learning':ti,ab,kw OR 'ensemble learning':ti,ab,kw OR 'artificial intelligence':ti,ab,kw OR 'random forest':ti,ab,kw OR 'neural network':ti,ab,kw OR 'neural networks':ti,ab,kw OR 'k-nearest neighbor':ti,ab,kw OR 'cnn':ti,ab,kw OR 'alexnet':ti,ab,kw OR 'vggnet':ti,ab,kw OR 'googlenet':ti,ab,kw OR 'support vector machine':ti,ab,kw OR 'svm':ti,ab,kw OR 'gradient boosting machine':ti,ab,kw OR 'nomogram':ti,ab,kw OR 'xgboost':ti,ab,kw OR 'adaboost':ti,ab,kw OR 'decision tree':ti,ab,kw OR 'resnet-50':ti,ab,kw OR 'resnet':ti,ab,kw OR 'naive bayesian':ti,ab,kw OR 'multilayer perceptron':ti,ab,kw OR 'bayesian network':ti,ab,kw | 339195 |
| #4 | 'radiomics'/exp | 9530 |
| #5 | (#1 OR #2) AND (#3 OR #4) | 1363 |

**4.Web of science**

| Search number | Query | Results |
| --- | --- | --- |
| #1 | TS=(Machine Learning OR Radiomics OR radiomic OR radiogenomic OR radiomics-based OR Texture OR Transfer Learning OR Deep learning OR Ensemble Learning OR artificial intelligence OR random forest OR neural network OR neural networks OR K-Nearest Neighbor OR CNN OR AlexNet OR VGGNet OR ResNet OR GoogLeNet OR Support vector machine OR SVM OR Gradient Boosting Machine OR Nomogram OR XGBoost OR Adaboost OR Decision tree OR ResNet-50 OR ResNet OR Naive Bayesian OR Multilayer perceptron OR Bayesian network ) | 1779438 |
| #2 | TS=(Rectal Neoplasms OR Cancer of Rectum OR Cancer of the Rectum OR neoplasm of the rectum OR neoplasma recti OR pararectal tumor OR pararectal tumour OR Rectal Cancer* OR rectal mass OR rectal neoplasia OR rectal neoplasm OR rectal tumor* OR rectal tumour OR Rectum Cancer* OR rectum mass OR rectum neoplasia OR rectum neoplasm* OR rectum tumor OR rectum tumour OR retrorectal tumor OR retrorectal tumour OR tumor of the rectum OR tumor recti OR tumour of the rectum OR tumour recti ) | 80299 |
| #3 | #7 AND #8 | 2057 |
